# Effects of a pelletized mash containing fresh *Asparagopsis taxiformis ‘brominata’* and oil on the performance of Holstein dairy cows

**DOI:** 10.1371/journal.pone.0335414

**Published:** 2025-11-13

**Authors:** Isabela Fonseca Carrari, Valerie Annabelle Achziger, Nathalia Veloso Tropia, Dhones Rodriguez de Andrade, Marcos Inacio Marcondes

**Affiliations:** 1 Department of Animal Sciences, Washington State University, Pullman, Washington, United States of America; 2 Department of Animal Sciences, Universidade Federal de Viçosa, Minas Gerais, Brazil; 3 William H. Miner Institute Agricultural Research Institute, Chazy, New York, United States of America; Universitas Sebelas Maret, INDONESIA

## Abstract

Enteric methane emissions from ruminants are a major environmental concern, and the use of *Asparagopsis taxiformis* has been proposed as a mitigation strategy. However, its effects on dry matter intake (DMI) and overall animal performance require further investigation. This study evaluated the impact of Brominata® pellets, a high-bromoform, low-iodine *Asparagopsis taxiformis* product, on the DMI, digestibility, milk production and composition, and feed efficiency of lactating Holstein cows. Twelve lactating Holstein cows (254 ± 54.2 days in milk) were assigned to one of two treatments: a control pellet or the Brominata® pellet. The study lasted 23 days, including a 7-day adaptation period, an 8-day ramp-up phase, and 8 days of full-dose feeding (0.25% DM). Cows were housed individually and provided with a total mixed ration and water. DMI was measured daily, and sorting behavior was assessed to determine if there was particle selection against the pellet. Milk yield was recorded electronically, and milk samples were collected during the last three days and analyzed for composition. Digestibility was evaluated using spot fecal collection during the last three days of trial. Data were analyzed using a randomized block design, with repeated measures applied where appropriate. Results indicated no significant differences in DMI, milk yield, or feed efficiency between treatments. Daily intake fluctuations were observed but were consistent across treatments, suggesting that environmental or random farm effects influenced intake rather than the inclusion of Brominata®. Sorting analysis showed no evidence of cows selectively avoiding or preferring the Brominata® pellets, indicating good palatability. Digestibility analyses revealed a significant reduction in fat digestibility in cows fed Brominata® (*P* = 0.046). Despite a reduction in fat digestibility, milk composition, including fat, protein, lactose, and solids non-fat, remained unaffected. In conclusion, including Brominata® pellets in the diet of lactating Holstein cows demonstrates no compromising effects on intake, milk production and composition, or feeding behavior.

## Introduction

Enteric methane (CH₄) emissions from ruminants, particularly cows, accounts for 30% of anthropogenic CH₄ emissions and 5% of the anthropogenic greenhouse gases (GHG) emissions in the world [1], and are a significant environmental concern, especially given the widespread use of these animals for milk and meat production. Antimethanogenic feed additives (AMFA) are shown as the most potent strategy for abatement of enteric CH_4_ emissions [2]. Among the proposed AMFA, *Asparagopsis taxiformis*, a red seaweed species rich in bromoform (CHBr_3_), has gained attention for its potent anti-methanogenic properties. Previous studies have demonstrated that *Asparagopsis taxiformis* or its extracts can significantly reduce enteric CH_4_ emissions in ruminants, with reported reductions of over 50% at moderate inclusion rates (e.g., 0.25% DM) without negatively affecting milk production [3,4]. However, higher inclusion rates (≥0.5% DM) have been associated with reduced dry matter intake (DMI) and milk yield [4,5], likely due to decreased palatability and potential ruminal disturbances [6,7]. Inconsistent effects on nutrient digestibility have also been observed, with reductions in fiber or fat digestibility [8,9] reported in certain contexts, potentially linked to shifts in rumen microbial populations induced by bromoform [10–12]. A meta-analysis [13] suggests that the efficacy of *Asparagopsis taxiformis* and tolerance by the animals are influenced by dose, form, and delivery method. Notably, unprocessed seaweed or mash forms have led to selective feed refusal and sorting behavior, particularly at high inclusion levels [6,14,15]. Additionally, excessive iodine content in certain seaweed species can lead to elevated milk iodine concentrations, raising concerns about food safety [16,17]. Several strategies have been proposed to address these challenges, and the development of pelleted, oil-enriched formulations with low iodine content, such as Brominata®, may help alleviate these challenges by improving palatability, stabilizing intake, and maintaining animal performance [18,19], thereby enhancing the practicality of *Asparagopsis taxiformis* adoption on commercial dairy farms [20,21].

A hypothesis yet untested is that *Asparagopsis spp.* alga material with high concentrations of minerals, particularly iodine, leads to refusals or selection against the seaweed, as proposed by Roque et al. [3]. This pilot study aimed to evaluate the impact of the Brominata® pellet (Blue Ocean Barns Inc., Kailua-Kona, Hawaii, USA), a high-bromoform/low-iodine pelletized fresh *Asparagopsis taxiformis* product, on DMI, digestibility, and cow performance. As one of the first investigations of pelletized *Asparagopsis taxiformis*, the results provide foundational knowledge to inform future research and offer insights into strategies for enhancing dairy cow productivity, safeguarding human health, and supporting environmental sustainability.

## Materials and methods

### Experimental design

The experiment was approved by the Institutional Animal Care and Use Committee (IACUC) at Washington State University under ASAF #7345. A power analysis was tested to estimate sample size for the preliminary response variables, including feed intake, milk yield, and feed efficiency, according to prior published literature values [6,22]. A sample size of 5 cows per treatment was projected as a sufficient sample size based on the power test analysis with α = 0.05 and power = 0.90 for the above-addressed variables [23]. Data from previous studies (not published) conducted by our research group were used to inform this calculation, particularly reflecting the low variability in dry matter intake (DMI) observed in lactation cows, with a coefficient of variation (CV) of approximately 14% [6,3]. Nonetheless, one additional cow was included per group to strengthen further the statistical power and account for potential dropout or variability. Thus, twelve lactating multiparous Holstein cows (254 DIM ± 54.2; 720 kg BW ± 49; 22.7 kg/d MY ± 0.62) were randomly assigned to one of two treatments: Control or Brominata® (containing *Asparagopsis taxiformis ‘brominata’*) and evaluated over 23 days, during which data on production, performance, and health were meticulously recorded.

### Housing and feeding

The cows were housed in individual box stalls and were individually fed a total mixed ration (TMR) formulated to meet the nutritional requirements of a Holstein cow, with an average mature body weight of 700 kg and a daily milk production of 25 kg/d ( [24]; Table 1). The feeding regimen allowed free access to water and a maximum of 5% of refusals (as-fed). Feed refusals were collected and weighed individually at 0700 of each day and compared to the previous day’s feed offered. If refusals exceeded 5%, the feed offered at the next feeding was reduced by 3%. If refusals were below 3%, the feed offered was increased by 3%. An initial 7-day adaptation period acclimated the cows to the experimental conditions, as they were already adapted to the diet. From days 8–16, a ramp-up period was implemented, during which the cows were gradually introduced to a 0.03% DM increasing inclusion of the pellet [control, as a placebo (brown algae base and same oil content and composition of the treatment pellet, but without bromoform) or Brominata®] eventually reaching the full dose of 0.25% DM (16 mg of bromoform per kilogram of dry matter intake). In the final 8 days of the trial, the cows were fed their lactating diet plus the final dose of the pellets, specific to their assigned treatment. The diet was initially prepared using a mixer truck wagon (International, 1991, Roto Mix 533−16, 7100). Once mixed, it was transferred to a Data Ranger (American Callan, 1989; scale model 1051), where the pellets were incorporated into the TMR and blended for 5 minutes immediately before feeding.

**Table 1:** Ingredients and chemical composition of the experimental diets.

Item	Control pellet	Brominata®pellet	Diet
Diet, % DM			
Alfalfa hay premium			19.53
Stem-rolled Corn			19.46
Corn silage			18.28
Concentrate			17.82
Dry-distilled grains			8.31
Triticale silage			6.65
Alfalfa hay common			5.82
Timothy hay			2.61
Cottonseed (with lint)			1.27
Pellet			0.25
Pellet^1^, %AsFed			
*Asparagopsis taxiforms* Biomass	0	13.1	
Kelp Powder^2^	33.0	0	
Water	37.0	39.4	
Vegetable/Seed Oil	23.7	25.2	
Carrier + Binder + Palatability Enhancer Mix^3^	6.3	22.3	
Composition, %DM unless otherwise stated			
Dry matter, % as-fed	55.30	61.60	46.62
Crude protein	4.90	12.30	18.42
ADF	20.70	6.40	21.44
aNDF	26.20	17.80	27.96
uNDF	5.20	26.30	10.14
Lignin	13.07	2.59	4.03
Starch	2.10	2.10	23.91
Fat	38.98	39.98	5.21
Residual organic matter	8.22	20.58	15.86
Ash	19.60	7.24	8.64
Ca	0.81	0.21	0.97
P	0.21	0.53	0.36
Mg	0.45	0.26	0.28
K	1.73	0.81	1.62
S	0.00	0.00	0.25
Iodine, mg/kg	652	830	3.30
Bromoform, mg/g of pellet	0	3.41 ± 0.19	0

^1^Full formulation is under proprietary restrictions.

^2^ *Ascophyllum nodosum.*

^3^Pellet binder, palatability enhancer, and wheat bran.

The ingredients of the pellets (Table 1) were combined in a mixer and blended for 5 min to ensure homogeneity of the mash. The mixture was subsequently processed using a cold-press pellet mill to produce pellets with a diameter of 5 mm and a length of 20 mm. Notably, no steam or heat was applied during the pelleting process, thereby minimizing potential degradation of bromoform and other heat-sensitive bioactive compounds, particularly in the treatment pellets. The pellets were stored under refrigeration throughout the trial to diminish the potential risk of mold growth on the control pellets. Although the Brominata® product can be stored at ambient temperature due to the antimicrobial properties of bromoform, both treatments were maintained under identical storage conditions to ensure consistency.

Our primary objective with the control pellet was to match the treatment pellet in iodine, oil, and dry matter content. Due to the minimal inclusion level, the slight differences in CP, ADF, and aNDF were considered negligible.

Although the exact concentrations of bromoform and iodine in each batch are proprietary (Blue Ocean Barns Inc., Kailua-Kona, Hawaii, USA) and were not publicly disclosed at the time of the study, the manufacturer provided estimated ranges based on historical batch analyses. Brominata® pellets contain 3.41 ± 0.19 mg of bromoform/g of pellet DM and 830 mg of iodine/g of pellet DM (Table 1), where each batch of pellets was analyzed to determine bromoform and iodine contents, ensuring consistency across batches and accuracy of inclusion levels. Bromoform from each batch of pellets was analyzed as follows: an aliquot of the sample was transferred into a microcentrifuge tube and centrifuged until the oil fraction was completely separated from the biomass or pellet product. The oil phase was carefully collected by pipette, transferred into a new microcentrifuge tube, weighed, and subsequently mixed with methanol, an internal standard, and a small quantity of beads. Samples were homogenized using a Tissuelyzer II and then centrifuged until complete phase separation of oil and methanol occurred. The methanol phase was collected and transferred into amber autosampler vials for GC–MS analysis. Gas chromatography–mass spectrometry was performed following a modified version of the “Rapid Analytical Method for the Quantification of Bromoform in the Red Seaweeds Asparagopsis armata and Asparagopsis taxiformis” described by [25].

### Sampling and sample analysis

Feces samples were collected every eight hours during the last three days of trial, and orts samples were collected before feeding time to perform a digestibility analysis. All individual samples were pre-dried at 55ºC in a forced-air oven for 72 hours, ground, and sifted through a 1-mm screen in a Wiley mill (Thomas Wiley®, Model 4, Philadelphia, PA, USA) and then a pooled sample was analyzed for DM, OM, CP, ether extract, NDF, and starch using near infrared spectroscopy (NIR) at Cumberland Valley Analytical Services (CVAS; Waynesboro, PA 17268). uNDF was analyzed through 240h *in vitro* incubation [26] at the same lab.

Milk production was electronically recorded daily using the DC305 milking software at Knott Dairy Center (KDC), Washington State University. Individual milk samples were collected from day 22–24, preserved with a bronopol tab, frozen at –20 ºC, and analyzed for fat, protein, lactose, and total solids with infrared spectroscopy (AOAC, 1990 [27]; method 972.160) at AgHealth Laboratories (Sunnyside, WA 98944). Somatic cell count (SCC) was also measured to detect any potential treatment effects on mammary gland health.

The pellets (Brominata® and Control) were analyzed for DM, OM, CP, ether extract, NDF, starch, and minerals (Ca, P, Mg, K, S) at AgHealth Laboratories (Sunnyside, WA 98944) accordingly to AOAC (2016) [28] 934.01, 942.05, 984.13, 920.39, 2002.04, 996.11, 985.01, 965.17, 985.01, 990.02, respectively.

Daily intake and sorting behavior were assessed to evaluate the palatability of the pellets. Intake was calculated as the difference between the amount of feed offered and the amount of orts (leftover feed) on a DM basis. Individual orts samples and TMR were collected from days 22–24, and the samples from the 3 days were pooled before analysis. Then, the pooled samples were analyzed for DM, OM, CP, ether extract, NDF, starch, and minerals (Ca, P, Mg, K, S) also using the NIR at Cumberland Valley Analytical Services (CVAS; Waynesboro, PA 17268). Iodine was analyzed at Eurofins SF Analytical Laboratories (New Berlin, WI 53151) accordingly to AOAC 2012.15 [29]. uNDF was analyzed at the same lab using 240h *in vitro* incubation [26]. The particle size distribution of the orts was determined by a particle analyzer, according to the American Society of Agricultural and Biological Engineers (ASABE) [30], to understand if the cows were sorting against or in favor of the pellets. A representative sample from the same orts sampled (∼100 g) was sieved for 10 min through a series of six screen sieves with nominal aperture sizes of 4, 2, 1.18, 0.71, 0.425, and 0.3 mm using a coarse sieve shaker (W.S. Tyler®, RX-812 model, Mentor, OH, USA). A bottom pan was included as a 7th screen to retain particles smaller than 0.125 mm. Each sieve was individually weighed before and after each sieve to obtain the weight of the samples on each sieve, thus determining particle size distribution. One run per sample was done, and sieves were cleaned thoroughly with an air compressor prior to each run.

### Statistical analysis

Data analysis was done using the GLIMMIX procedure of SAS (Statistical Analysis System, version 9.4). All variables were analyzed as a randomized block design with 6 replications and two blocks – side of the barn (6 animals per side), following the model:


$$Y_{ij}\;=\;\mu \;+\;T_{i}\;+B_{j}\;+\epsilon_{ij}\mathrm{\backslash ]}$$

Where μ = general mean; T_i_ = fixed effect of the treatment i; Bj = random effect of block j; ε_ij_ = random error.

**Fig 1:**
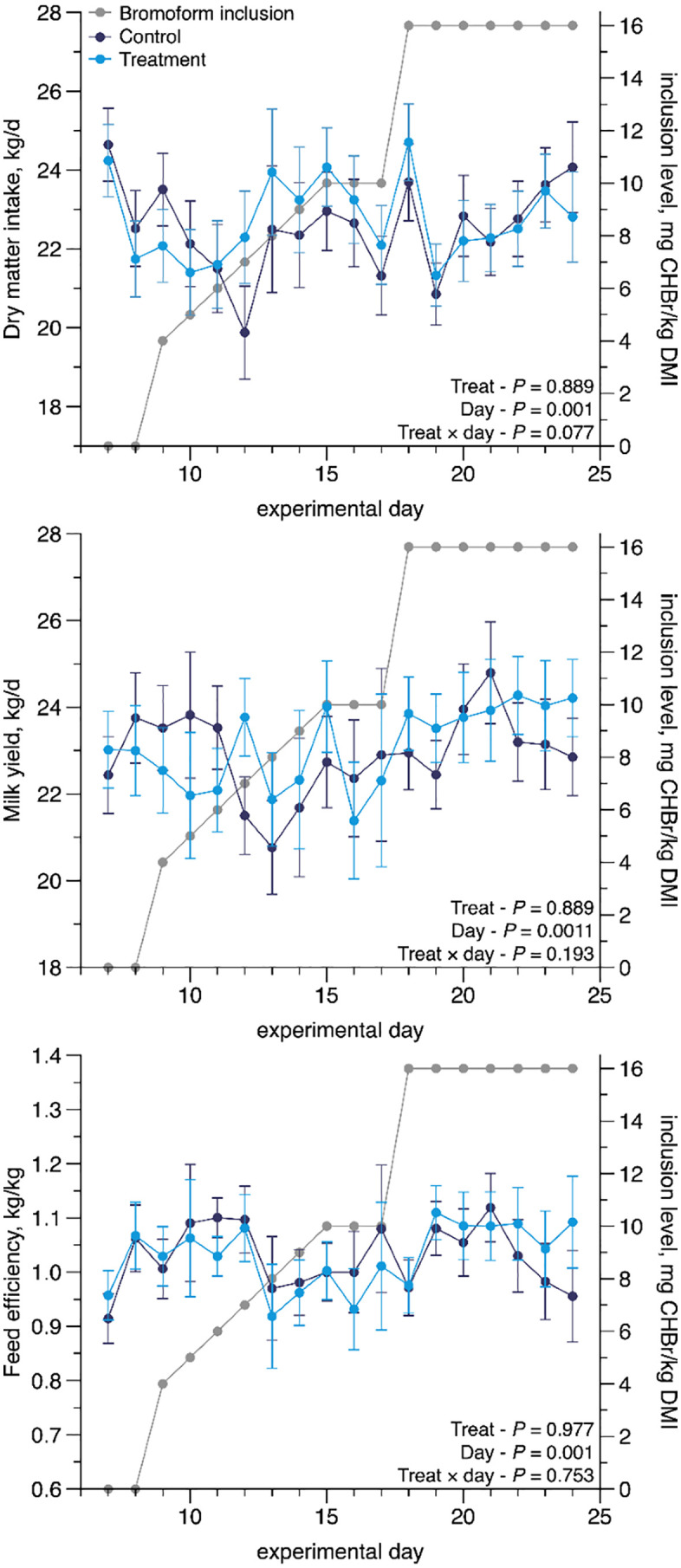
Effect of Brominata® on intake, milk composition, and feed efficiency of lactating Holstein cows throughout 24 days.

The barn was divided into two sides, with the milking parlor located centrally. As a result, cows were housed in two distinct environments that differed in several environmental factors, including air flow patterns, ambient temperature, and exposure to natural sunlight. These differences had the potential to influence cow behavior, feed intake, and performance. Therefore, barn side was included as a blocking factor in the experimental design to account for this potential variation and improve the precision of treatment comparisons. It is important to note that although environmental conditions varied between sides, the physical layout was standardized: all 12 pens were identical in size, design, and orientation, ensuring consistency in space allowance, bunk access, and bedding across treatments. The average of measurements from days 6 and 7 (end of the adaptation period) was used as the covariate for all analyses. However, the covariates were excluded from the model in all cases due to P > 0.05. DMI, milk yield, and feed efficiency were also evaluated throughout the entire experiment, with day considered repeated measure. The analysis used daily data as repeated measurements or data pooled over three-day intervals as repeated measures. Regardless of the approach, no significant day-by-treatment interaction or treatment effects were observed for DMI, milk production, or feed efficiency (P > 0.05, Fig 1). While daily analysis of intake or production is not typically performed, we opted to include it in this study due to prior reports indicating a potential drop in DMI and subsequently in milk yield when cows begin consuming seaweed [3]. This analysis aimed to identify potential fluctuations in intake or production during the ramp-up phase or full-dose application of the treatments. Statistical significance was declared at P ≤ 0.05, and the threshold for trends was set at 0.05 < P ≤ 0.10.

## Results and discussion

This pilot study was designed to evaluate the impact of the Brominata® pellet, a high-bromoform/low-iodine pelletized *Asparagopsis taxiformis* product, on DMI, nutrient digestibility, and overall cow performance. As one of the first studies to investigate a pelletized form of *Asparagopsis taxiformis*, it provides foundational knowledge on the potential of this feed additive to be incorporated into practical dairy production systems. The findings contribute to a growing body of evidence supporting the use of seaweed-based supplements as tools to enhance animal productivity, improve feed efficiency, and promote environmental sustainability. Importantly, this study also highlights key areas requiring further investigation.

*Ascophyllum nodosum* was selected as the “control” seaweed ingredient because it provides iodine but lacks antimethanogenic activity. As demonstrated by [31], an in vitro comparison of *Asparagopsis taxiformis* and *A. nodosum* showed no significant reduction in methane production for *A. nodosum*. With respect to bromoform concentrations, freshly harvested *A. nodosum* contains negligible levels compared with *Asparagopsis taxiformis*. [32] reported bromoform concentrations of approximately 8 µg/kg in *A. nodosum*, which is about six orders of magnitude lower than the levels commonly detected in *Asparagopsis taxiformis*. Consistent with these findings, GC–MS analysis of the processed *A. nodosum* used in the present study confirmed non-detectable levels of bromoform.

The expected treatment effect was based on findings from [3], who reported a 37.8% reduction in DMI associated with a 50% reduction in methane emissions. Given that *Asparagopsis taxiformis* is proposed to reduce methane by a similar magnitude, we used this relationship as a basis for estimating the potential impact on DMI. However, to remain conservative, we used a projected 30% reduction in intake as the target difference for power estimation.

### Dry matter intake

Daily intake patterns were analyzed to detect short-term fluctuations in response to Brominata® inclusion. While a transient decrease in intake was visually observed in the treatment group on day 19 (following a spike on day 18; Fig 1), a similar pattern occurred in the control group. No significant treatment × time interaction or main treatment effects were detected (P > 0.05), suggesting these fluctuations possibly reflected random farm-related events (e.g., environmental variation) rather than treatment effects.

Consistent with this, mean DMI, organic matter intake (OMI), and nutrient intake did not differ between treatments (P > 0.05; Table 2). This contrasts with several prior studies in which *Asparagopsis taxiformis* supplementation reduced DMI. For instance, [6] observed a dose-dependent reduction in DMI when lactating dairy cows were fed *A. taxiformis* at inclusion rates of 0.12%, and 0.25% DM. [7] reported that DMI in sheep declined progressively as *A. taxiformis* inclusion increased from 0.1% to 1.0% DM, suggesting a potential palatability or ruminal effect.

**Table 2:** Effect of Brominata® on intake, digestibility, and performance of lactating Holstein cows.

Item	Control	Brominata®	SEM	P-value
Intake, kg/d				
Dry matter	21.93	22.02	1.078	0.946
Organic matter	20.05	20.12	2.337	0.949
Crude protein	4.59	4.48	0.229	0.615
Neutral detergent fiber	6.96	6.81	0.342	0.641
Starch	5.96	5.82	0.297	0.630
Fat	1.29	1.26	0.064	0.624
Residual organic matter	4.03	3.94	0.201	0.603
Digestibility, g/g				
Dry matter	0.72	0.70	0.007	0.134
Organic matter	0.72	0.71	0.006	0.175
Crude protein	0.76	0.72	0.010	0.056
Neutral detergent fiber	0.53	0.51	0.016	0.304
Starch	0.97	0.97	0.002	0.335
Fat	0.90	0.86	0.012	0.046
Residual organic matter	0.77	0.75	0.009	0.375
Production				
Milk yield, kg/d	23.06	24.17	0.586	0.398
Feed Efficiency, kg/kg	0.99	1.07	0.072	0.411
Fat, %	4.62	4.70	0.427	0.897
Protein, %	3.28	3.35	0.215	0.674
Lactose, %	4.46	4.50	0.116	0.828
Solids non-fat, %	7.74	7.84	0.301	0.713
Log SCC^2^	1.24^b^	2.25^a^	0.262	0.021
MUN^1^, mg/dL	14.38	12.70	0.937	0.233

^1^Milk urea nitrogen.

^2^Somatic Cell Count.

According to [24], the daily iodine requirement for lactating dairy cows is 10.35 mg. In the present study, the basal TMR contained 3.3 mg/kg DM of iodine (Table 1). The inclusion of Brominata® pellets increased the iodine concentration to 5.37 mg/kg DM, while the TMR containing control pellets provided 4.92 mg/kg DM. Both diets exceeded the recommended requirement but remained well below the maximum tolerable concentration of 50 mg/kg DM established by [24].

### Nutrient digestibility

Brominata® supplementation significantly decreased fat digestibility (P = 0.042), with a trend toward reduced crude protein digestibility (P = 0.056; Table 2). This reduction in fat digestibility was unexpected, as both treatment and control pellets contained comparable fat concentrations (~39%; Table 1), and fat intake did not differ between groups (P = 0.624). While few studies have reported effects of *Asparagopsis taxiformis* on fat digestibility, [8] observed shifts in milk short and medium fatty acid profile when cows were fed *Asparagopsis taxiformis*, suggesting possible interactions with lipid metabolism and digestion. The precise mechanisms remain unknown but may involve ruminal microbial changes or bromoform’s effects on biohydrogenation pathways. Bromoform and other halogenated compounds, such as those found in *Asparagopsis* species, have been shown to alter rumen microbial populations, including those involved in lipid metabolism. While our study was not designed to isolate these microbial changes, the reduction in fat digestibility we observed is consistent with these known effects of *Asparagopsis*-derived compounds on rumen fermentation dynamics.

No significant effects were observed on the digestibility of DM, OM, or fiber fractions (P > 0.05), but literature reports variable effects on nutrient digestibility. [3] reported that high levels of *Asparagopsis taxiformis* supplementation (0.5% DM) reduced both DM and NDF digestibility in beef heifers, likely linked to shifts in rumen fermentation patterns. These findings suggest that effects on digestibility depend on both dose and delivery method; the lower inclusion rate used here was likely smaller than the threshold (> 0.5% inclusion in the DM) [4–7] where major adverse effects occur, aside from the fat digestibility response. Importantly, reductions in methane emissions observed in these studies are not fully explained by changes in digestibility alone. Rather, bromoform directly inhibits methanogenic archaea [12], leading to lower methane output even in the absence of major shifts in overall digestibility or total volatile fatty acid (VFA) profile.

### Feed sorting behavior

Feed sorting analysis indicated no preference for or against the treatment pellets (Table 3). Sorting behavior also remained consistent between the end of the ramp-up period (day 16) and the full inclusion phase (days 22–24; P > 0.338), indicating that cows adapted well to the pellets and did not exhibit aversive responses.

**Table 3:** Effect of Brominata® on sorting of lactating Holstein cows.

DAY	16		23		24		25		SEM	P-value		
Item	TR	CON	TR	CON		TR	CON	TR	CON	TR	PER	TxP
4.00 mm	42.87	48.03	52.36	46.57	32.30	30.42	64.99	72.22	4.177	0.726	0.001	0.393
2.00 mm	29.14	26.71	25.12	26.27	27.94	28.46	23.17	17.15	2.501	0.484	0.005	0.338
1.18 mm	12.61	11.51	9.14	10.23	17.98	19.43	5.85	4.74	1.341	0.930	0.001	0.660
710 μm	11.06	9.21	9.25	11.30	15.52	15.47	4.42	4.68	1.148	0.898	0.001	0.673
425 μm	4.11	4.42	4.06	5.56	6.06	6.04	1.50	1.16	0.820	0.580	0.001	0.580
300 μm	0.16	0.09	0.05	0.06	0.14	0.13	0.05	0.03	0.030	0.181	0.031	0.562
Bottom	0.05	0.04	0.03	0.02	0.04	0.05	0.01	0.03	0.010	0.854	0.048	0.592

An important aspect of this study is that the *Asparagopsis taxiformis* used was provided as a commercially formulated pellet containing both seaweed and a substantial amount of oil added to the mash during pellet production. The use of a pelleted product offers practical advantages for on-farm feeding and may improve palatability and intake consistency compared to loose or mash forms of *Asparagopsis taxiformis* [18]. Prior studies show that unprocessed seaweed can negatively affect feed palatability and cause selective refusal and reduced intake due to strong odor, unpalatable taste, or high mineral content (particularly iodine), especially at higher inclusion rates [3,14]. The inclusion of oils in the pellet matrix may help mask the taste and odor of bromoform-containing compounds, reducing aversive responses and supporting more stable DMI and feeding behavior, since fat can act as a carrier for lipophilic compounds like bromoform, potentially modulating their release and reducing any immediate negative effects on taste or rumen environment. [18,19]. Moreover, the oil matrix may also influence the release dynamics of bromoform in the rumen, potentially modifying its effects on methane emissions and fermentation pathways. The present results (no negative effects on DMI, no feed sorting, and stable milk production) suggest that the pelleted and oil-enriched formulation of Brominata® is effective at maintaining palatability and animal performance, even when delivering bioactive levels of bromoform. It is also important to note that the Brominata® pellets used here were low in iodine, addressing one of the hypothesized causes of feed refusal seen in other trials using *Asparagopsis taxiformis* [3]. By limiting iodine exposure, the risk of mineral imbalances or off-flavors associated with excessive iodine intake was likely reduced.

Some studies have shown that *Asparagopsis taxiformis* in mash or loose powder forms often led to feed refusal or sorting behavior, particularly at higher inclusion levels (around 1% of DM) [6,15,23]. The pellet formulation used here likely improved palatability and acceptance, helping explain the absence of negative effects on intake or sorting in this study. These findings emphasize the importance of delivery method and product formulation when evaluating the use of *Asparagopsis taxiformis* in dairy diets.

### Milk production and composition

Milk yield and composition were unaffected by treatment (P > 0.05 for milk yield, protein, fat, lactose, solids non-fat, and milk urea nitrogen; Table 1). [3] showed that methane reductions of ~50% could be achieved without compromising milk production at inclusion rates of 0.25% DM; however, they noted that milk yield reductions occurred at inclusion levels of 0.5% DM.

In the present study, maintenance of DMI likely supported stable milk production. However, an unexpected increase in milk SCC was observed in the treatment group. The cause of this increase remains unclear, as no clinical mastitis cases or other confounding factors were detected during the trial. While prior studies with *Asparagopsis taxiformis* have not consistently reported differences in SCC, some authors suggest that seaweed-derived bioactives may modulate immune responses. [27,28] reviewed evidence that polysaccharides and phenolic compounds in seaweeds can influence both innate and adaptive immunity in animals. [29] reported that seaweed supplementation decreases the abundance of *Staphylococcus* spp. bacteria (including *Staphylococcus aureus*) in the milk of dairy cows, which could affect SCC values. Whether such effects occur with *Asparagopsis taxiformis* supplementation in dairy cows remains to be determined and warrants further investigation.

This short-term pilot study was conducted to evaluate the effects of a seaweed-based product on DMI, nutrient digestibility, and dairy cow performance. As initial findings, the absence of significant effects on intake, digestibility, and milk yield or composition suggests that the product is safe for inclusion in dairy cow diets. Methane emissions were not directly measured, which prevented conclusions regarding the extent of enteric methane mitigation, a primary objective of *Asparagopsis taxiformis* supplementation. Additionally, no analyses were conducted on bromoform or iodine residues in milk, leaving uncertainty about the potential implications for food safety and human health. These limitations underscore the need for follow-up studies that integrate comprehensive assessments of both environmental and food safety endpoints. Taken together, the current results provide valuable insights into the feasibility of supplementing with pelletized *Asparagopsis taxiformis*, while also emphasizing the importance of addressing these gaps to ensure its safe and effective adoption in commercial dairy production.

Although methane emissions were not measured in this study, it is reasonable to assume that mitigation effects may have occurred, given the known properties of *Asparagopsis taxiformis*. Indeed, a subsequent full-lactation trial conducted by our group (unpublished) after the successful results of this study demonstrated reductions in enteric methane emissions when animals received the same product. Together, these results support the safety and potential efficacy of pelletized *Asparagopsis taxiformis* supplementation, while also highlighting the importance of future studies that integrate longer-term performance, methane quantification, and residue analysis to comprehensively evaluate its role in sustainable dairy production. Nevertheless, future studies should also directly measure milk bromoform and iodine residues to confirm product safety and ensure compliance with human health guidelines.

## Conclusion

This pilot study demonstrated that supplementing lactating Holstein cows with Brominata® had no adverse effects on dry matter intake, milk production, feed efficiency, or milk composition. Feed sorting behavior remained unchanged, indicating good palatability and acceptance of the pellet. While fat digestibility was significantly reduced and a trend toward lower protein digestibility was observed, these changes did not affect animal performance. The unique formulation of Brominata®, including its pelleted form and high oil content, may have contributed to maintaining intake and feeding consistency, contrasting with previous reports of reduced palatability.
